# Phosphate-Linked Silibinin Dimers (PLSd): New Promising Modified Metabolites

**DOI:** 10.3390/molecules22081323

**Published:** 2017-08-11

**Authors:** Valeria Romanucci, Raffaele Gravante, Martina Cimafonte, Cinzia Di Marino, Gilles Mailhot, Marcello Brigante, Armando Zarrelli, Giovanni Di Fabio

**Affiliations:** 1 Department of Chemical Sciences, University of Naples “Federico II”, Via Cintia 4, I-80126 Napoli (NA), Italy; valeria.romanucci@unina.it (V.R.); raffaele.gravante@unina.it (R.G.); m.cimafonte@hotmail.it (M.C.); cdimarin@unina.it (C.D.M.); zarrelli@unina.it (A.Z.); 2 Consorzio Interuniversitario Sannio Tech, P.zza San G. Moscati 8, SS Appia km 256, 82030 Apollosa (BN), Italy; 3 Institut de Chimie de Clermont-Ferrand, CNRS, SIGMA Clermont, Université Clermont Auvergne, F-63000 Clermont–Ferrand, France; gilles.mailhot@uca.fr (G.M.); marcello.brigante@uca.fr (M.B.)

**Keywords:** silibinin, phosphodiester, oligoflavonoids, xanthine/xanthine oxidase assay, radical scavengers, reactive oxygen species (ROS)

## Abstract

By exploiting the regioselective protection of the hydroxyl groups of silibinin along with the well-known phosphoramidite chemistry, we have developed an efficient strategy for the synthesis of new silibinin-modified species, which we have named Phosphate-Linked Silibinin Dimers (PLSd), in which the monomer units are linked by phosphodiester bonds. The antioxidant abilities of the new PLSd were estimated on HepG2 cells using DPPH free radical scavenging and xanthine/xanthine oxidase assays. The new phosphate-metabolites showed a higher anti-oxidant activity than the silibinin, as well as very low toxicity. The ability to scavenge reactive oxygen species (ROS) such as singlet oxygen (O21) and hydroxyl radical (${\mathrm{HO}}^{•}$) reveals that the two dimers are able to scavenge ${\mathrm{HO}}^{•}$ about two times more effectively than silibinin. Finally, solubility studies have shown that the PLSd present good water solubility (more than 20 mg·L^−1^) under circumneutral pH values, whereas the silibinin was found to be very poorly soluble (less than 0.4 mg·L^−1^) and not stable under alkaline conditions. Together, the above promising results warrant further investigation of the future potential of the PLSd as anti-oxidant metabolites within the large synthetic polyphenols field.

## 1. Introduction

**Figure 1:**
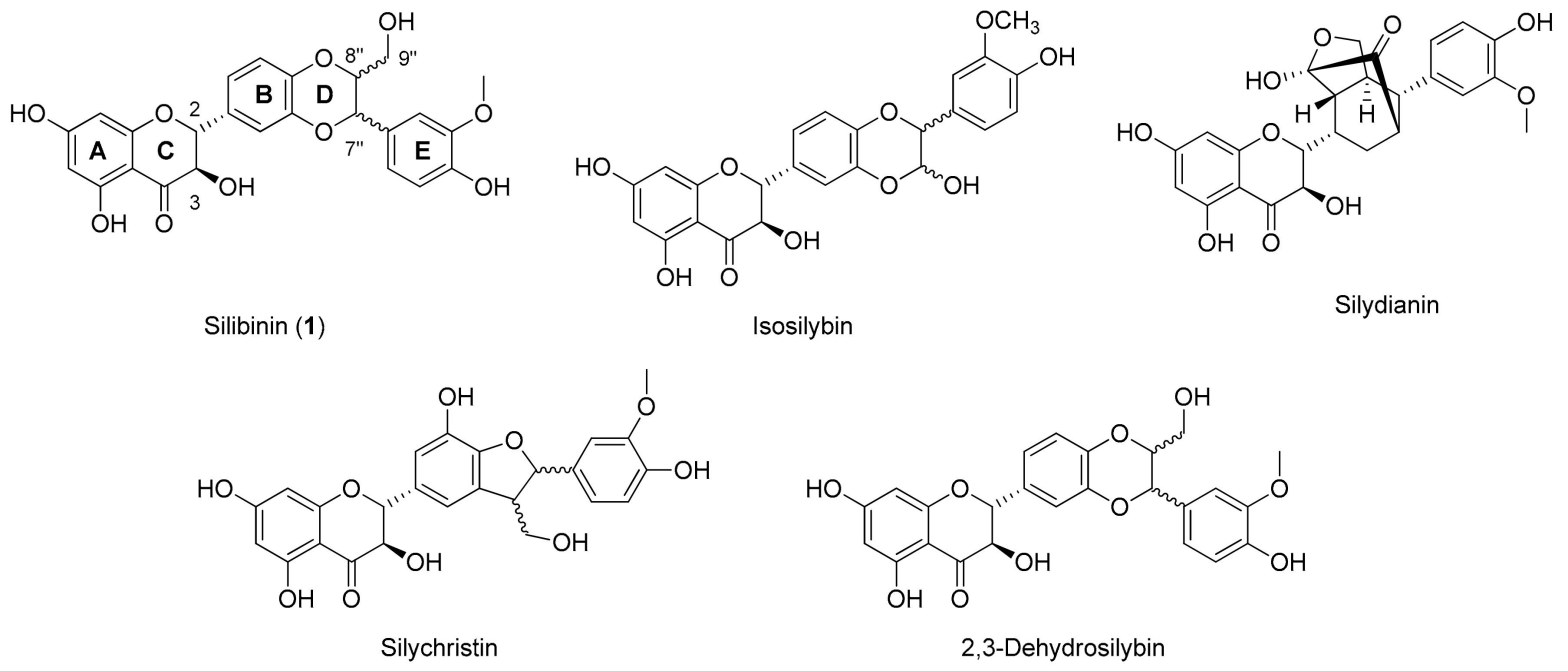
Structures of all components of silymarin.

Polyphenols are widely distributed in Nature and in many cases are the active compounds of the medicinal plants from which they can be isolated. Despite their few and common biosynthetic origins, polyphenols encompass many subclasses of structurally diverse entities from different pharmacological properties. Most of them are small molecules bearing a few hydroxyphenyl units, but many others consist of more complex oligomeric polyphenols with interesting pharmacological properties. The high structural diversity of these oligomers, attributed to regio- and stereochemical variations of their inter-linkages, makes their purification from natural extracts very laborious. For the same reasons, the design and chemical synthesis of these oligomers is a difficult challenge. Many studies suggest that the mechanisms by which plant polyphenols exert their protective actions against cardiovascular and neurodegenerative diseases, as well as cancer and diabetes, are due to their reactive oxygen species (ROS) scavenging activities as well as their ability to directly bind to biotargets such as peptides, proteins and nucleic acids [1]. The acclaimed capability of polyphenols to scavenge reactive oxygen species (ROS) is frequently cited to be the key property underlying the prevention and/or reduction of oxidative stress-related chronic diseases and age-related disorders such as cardiovascular diseases, carcinogenesis, and neurodegeneration [2,3,4,5]. Silibinin (**1**, Figure 1), a flavonolignan derived from the combination of a phenylpropanoid (taxifolin) and coniferyl alcohol, belongs to this large group of polyphenols [6]. Silibinin is the most abundant component of silymarin, the extract matrix of the milk thistle seeds (*Silybum marianum* (L.) Gaertn.) [7]. The silymarin extract contains a variety of the flavonolignans as indicated in Figure 1.

In the past two decades, the silibinin has received much attention [8] due to its anticancer and chemopreventive actions, as well as its hypocholesterolemic [9], cardioprotective and neuroprotective [10,11,12] activities. Silibinin is a diastereoisomeric mixture of two flavonolignans, namely silybin A and silybin B, at a ratio of approximately 1:1. In vivo applications of silibinin are however hampered by its very low bioavailability and water solubility.

The synthesis of 3,9′′-*O*-bis-hemisuccinate silibinin, was the first example of a water-soluble analogue that enabled an intravenous application of silibinin (Legalon-SIL, Madaus, Germany) for the treatment of acute liver intoxication by mycotoxins. In an attempt to improve its biological properties and facilitate in vivo applications, a variety of structural modifications have been proposed, however, new modifications are still few in number [8]. Therefore, new synthetic approaches for selectively modifying silibinin are of high interest.

Natural products, including flavonolignans, terpenes and lignans are the focus of our latest research [13,14,15,16,17] and in this framework the synthesis of new 9′′-phosphodiester conjugated silibinin derivatives is noteworthy [18,19,20]. The insertion of a suitable label and a phosphate group increases the water solubility of the silibinin aglycon and promotes a pro-drug approach making it possible to improve its pharmaceutical, pharmacokinetic and/or pharmacodynamic properties.

**Scheme 1:**
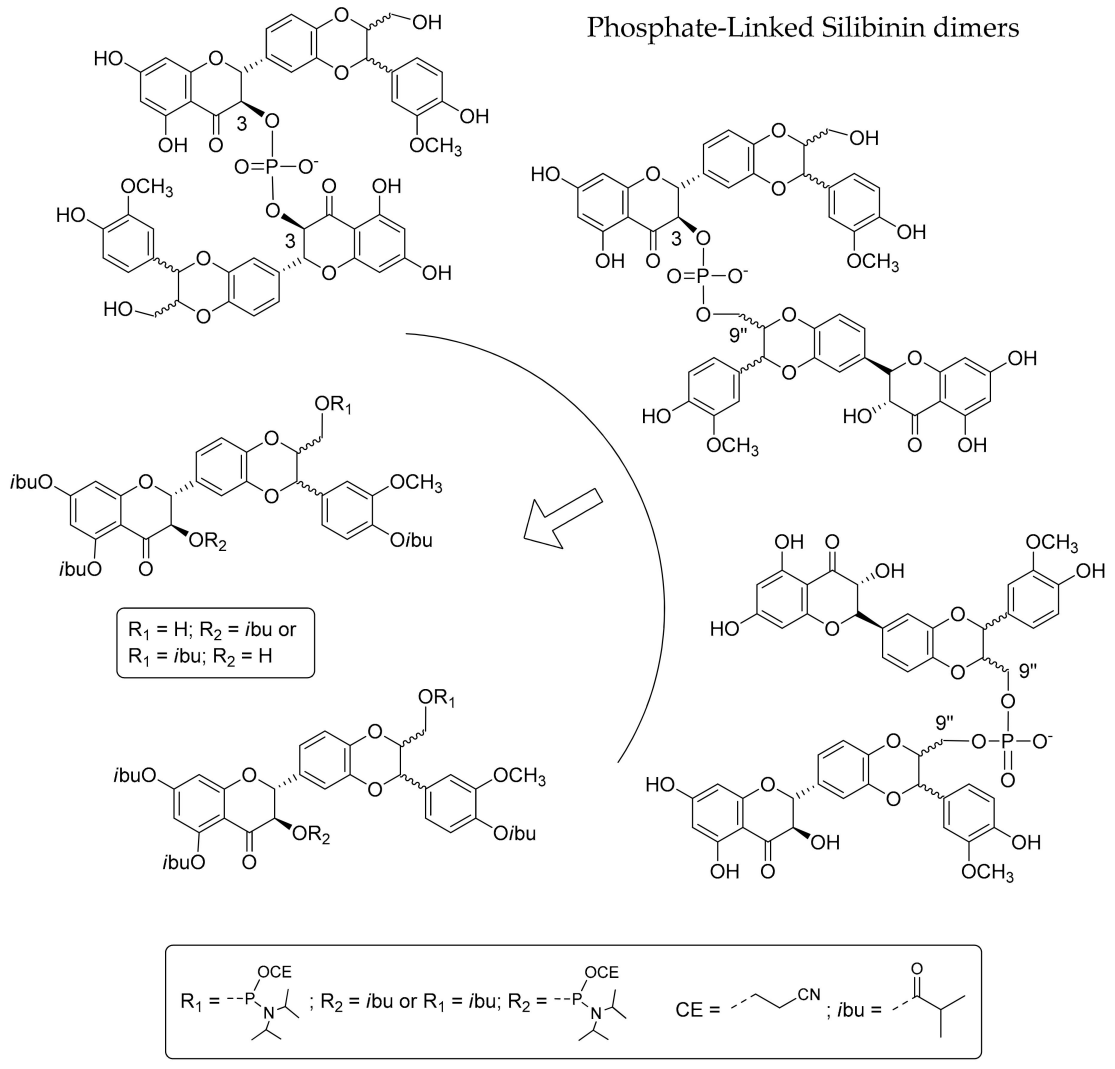
Retrosynthetic scheme of Phosphate-Linked Silibinin dimers.

As a part of our continuing research effort on the synthesis of new natural product analogues by exploiting phosphoramidite chemistry [21,22,23,24] and inspired by oligoflavonoid structures [1], here, we present an efficient synthesis of some new silibinin derivatives, the Phosphate-Linked Silibinin dimers (PLSd) and related studies of their redox behaviour. In particular, exploiting the selective protection of the hydroxyl groups of the silibinin, we have developed an efficient strategy for the synthesis in good yields of new 3-3, 3-9′′ and 9′′-9′′ silibinin dimers (Scheme 1).

Preliminary experiments were carried out to evaluate some properties by different approaches (radical scavenger behaviour and oxidative stress protection) and compare them to those of the parent silibinin. For use in a pro-drug approach, the serum stability of the new dimers was determined by HPLC analyses, and their anti-oxidant behaviours were evaluated on HepG2 cells by the xanthine/xanthine oxidase (X/XO) assay. The water solubility, the radical scavenger efficiency (DPPH test) and the ability to react with reactive oxygen species (ROS) of silibinin and the dimers were also determined. Reactivity with ROS was quantified by estimating their second order rate constant with singlet oxygen (O21) and hydroxyl radical (${\mathrm{HO}}^{•}$) in solution. For this purpose, 545 nm centered excitation of Rose Bengal (RB) and laser flash photolysis experiments were coupled with a kinetic competition approach.

## 2. Results and Discussion

### 2.1. Synthesis of Phosphate-Linked Silibinin Dimers

**Scheme 2:**
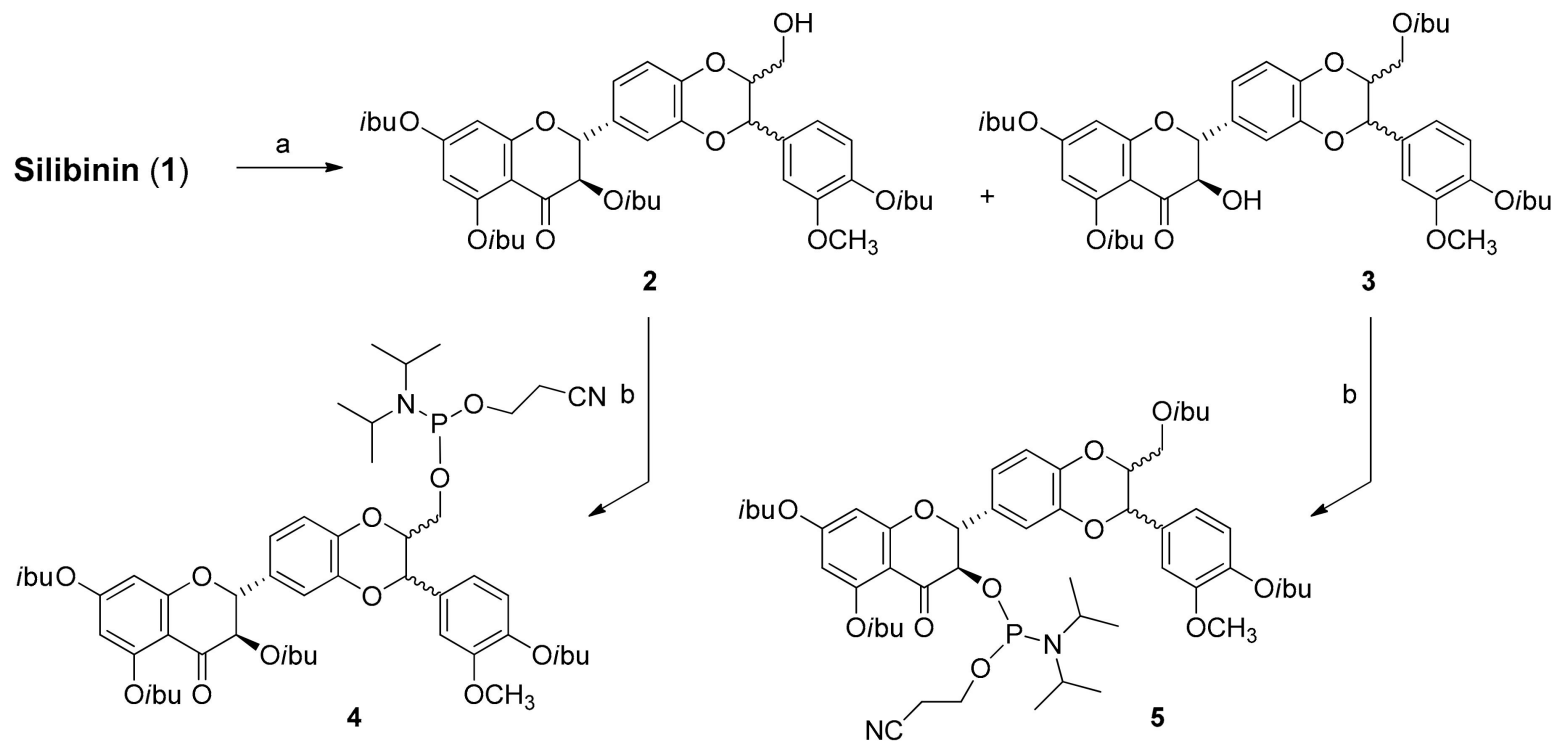
Synthesis of silibinin building blocks*. Reagents and conditions*: (a) isobutyryl chloride, DIEA, THF 0 °C; (b) 2-cyanoethyl-*N*,*N*-diisopropylaminochlorophosphoramidite, DIEA, DCM, rt.

Silibinin is a flavonolignan with five hydroxyl groups (three phenolic, one secondary and one primary) with different reactivity. To obtain suitable building blocks **2** and **3**, we developed regioselective reactions using different amounts of isobutyryl chloride in THF to 0 °C (Scheme 2). After 30 min, the reactions were stopped by quenching with a few drops of H_2_O and after silica gel chromatography, the desired compounds **2** and **3** were obtained in 15% and 28% yields, respectively. To prepare the corresponding phosphoramidites, we converted compounds **2** and **3** (Scheme 2) into the phosphoramidites **4** and **5** by the classic reaction with 2-cyanoethyl-*N*,*N*-diisopropylamino-chlorophosphoramidite.

After appropriate work-up, silica gel chromatography afforded the desired compounds **4** and **5** at 65% yields. The identities of compounds **2**–**5** were confirmed by NMR (^1^H, ^13^C and ^31^P) and ESI-MS data.

**Scheme 3:**
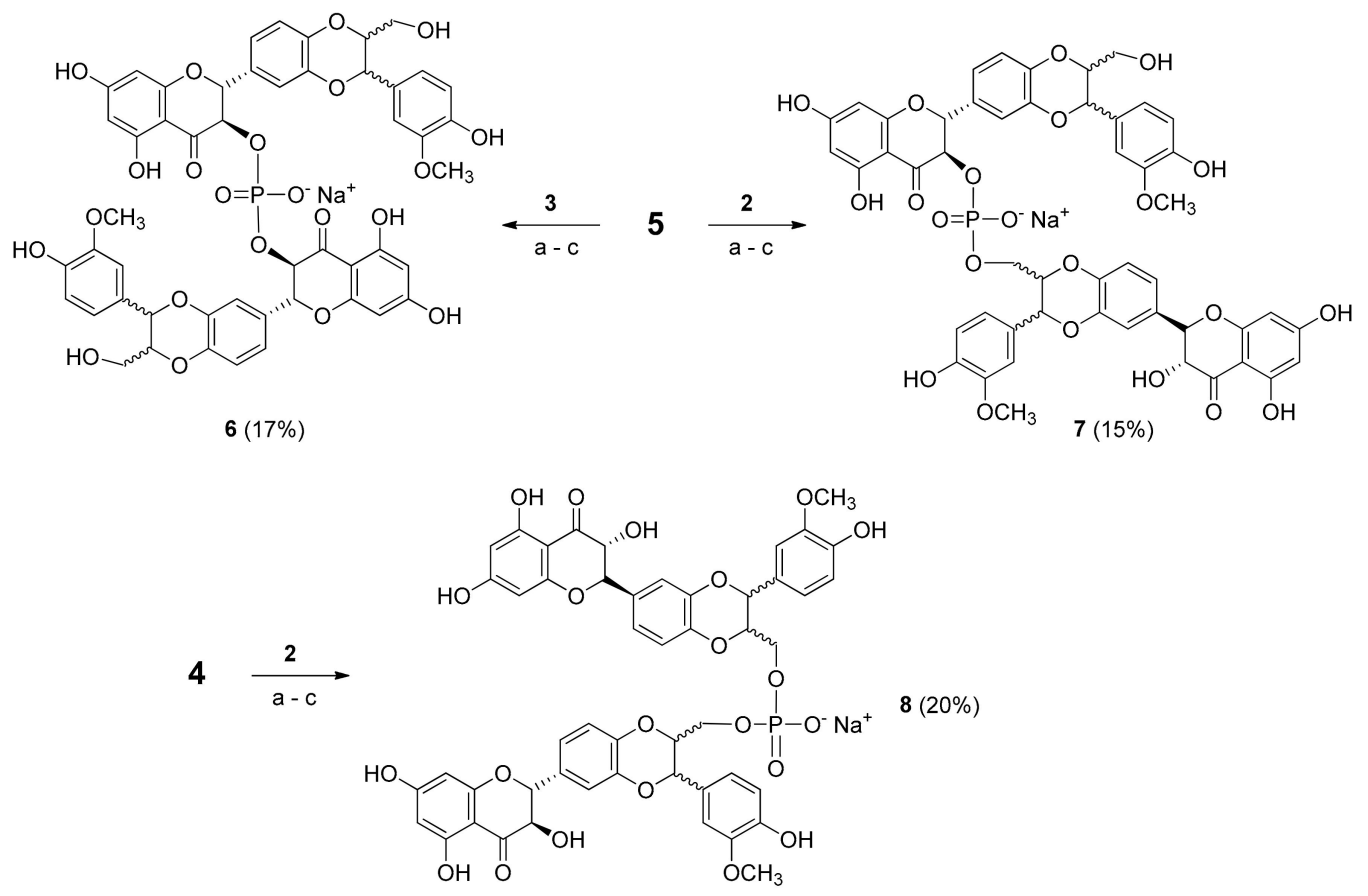
Synthesis of Phosphate-Linked Silibinin dimers **6**–**8**, *Reagents and conditions*: (a) 0.45 M DCI, DCM, rt (b) 5.5 M TBHP in decane, rt (c) 28% NH_4_OH aqueous solution and CH_3_OH (1:1, *v*:*v*), 50 °C.

Compounds **2** and **3** were coupled with phosphoramidites **4** and **5** in the three different prositions using 0.45 M DCI (Scheme 3).

After the oxidative treatment with 5.5 M *tert*-butyl hydroperoxide (TBHP) solution in decane, the crude material was purified by gel chromatography. Several complex fractions were recovered, but only those with a ^31^P-NMR signal were treated with a 28% NH_4_OH aqueous solution and CH_3_OH (1:1, *v*:*v*) at 50 °C, allowing the full deprotection from isobutyric and cyanoethyl groups. After RP-18 HPLC analyses and purification, the products were converted into the corresponding sodium salts by cation exchange on a DOWEX (Na^+^ form) resin, leading to the desired phosphodiester derivatives **6**–**8** in good yields (17%, 15% and 20%, respectively).

As expected, the NMR spectra of the new dimers look like those of a mixture of compounds (a diastereoisomer mixture), so unfortunately a more detailed analysis was not possible. The products obtained by cation exchange as sodium salts were suspended in DMSO-*d*_6_, but the NMR spectra did not allow a detailed assignment of the different spin systems. Dimers **6**–**8** were finally characterized by ^31^P-NMR and MALDI-MS (Table 1).

### 2.2. Antioxidant Activity: DPPH Test

The DPPH test is a non-enzymatic method commonly used to provide basic information on the scavenging potential of stable free radicals in vitro. In a preliminary study, the new derivatives were subjected to a DPPH free radical scavenging assay (Table 1), and for comparison purposes, the antioxidant activity of silibinin and quercetin were evaluated as controls. In all cases, the radical scavenging activities of the dimers **6**–**8** were better than that of silibinin (**1**).

### 2.3. Cytotoxicity (MMT Test) and Protective Activity (X/XO Assay)

**Figure 2:**
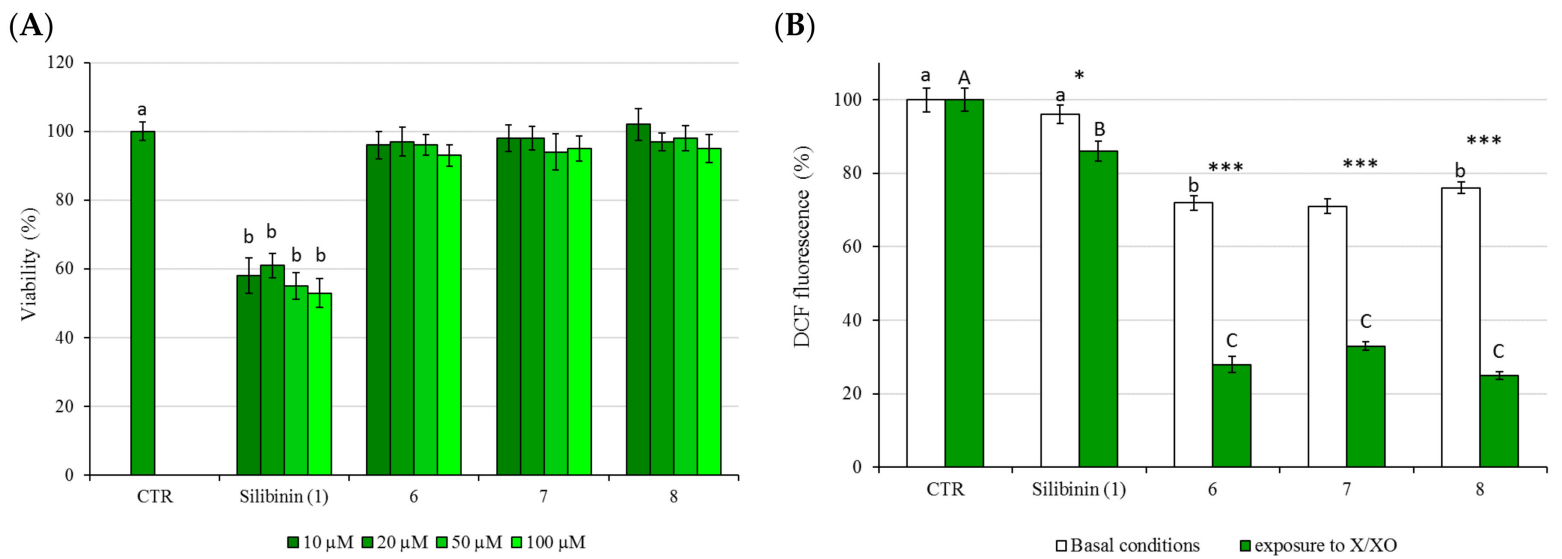
(**A**) The effects of 24 h treatment on HepG2 cell viability as determined by MTT assay. Different letters indicate significant differences among control and different concentrations (One way ANOVA, *p* < 0.05); (**B**) Protection against XO-induced intracellular ROS generation: DCF fluorescence was measured in basal conditions (white bars) or following cells exposure to X/XO system for 3 h (green bars). The cells were pretreated with each dimer (**6**–**8**) at concentrations of 20 μM. The values are expressed as % compared to the fluorescence value observed in control untreated cells which was set at 100. Asterisk indicates significant differences between basal conditions and exposure to X/XO, *: *p* < 0.05, ***: *p* < 0.001; different letters indicate significant differences among control, silibinin (**1**), **6**, **7** and **8** within basal conditions and exposure to X/XO (Two way ANOVA).

The new silibinin dimers **6**–**8** were non-cytotoxic to HepG2 cells, with cell viabilities exceeding 90% at concentrations that higher than 10 μM (20, 50 and 100 μM) and at 24 h of exposure (Figure 2A). The new compounds were also non-cytotoxic, with cell viabilities exceeding 90% after 48 h exposure (data not shown). The antioxidant activity of the dimers **6**–**8** was evaluated using HepG2 cells as models and the 2′,7′-dichlorofluorescin diacetate (DCFH-DA) test.

In this assay, intracellular reactive oxygen species (ROS) levels were measured by the oxidative conversion of stable, non-fluorescent DCFH-DA to the highly fluorescent 2′,7′-dichlorofluorescein (DCF) occurring in the presence of ROS [25,26]. In particular, DCFH-DA easily crosses cell membranes and is transformed to non-fluorescent DCFH via a hydrolysis reaction catalysed by intracellular esterases. Reactions between DCFH and intracellular ROS lead to a highly fluorescent product, dichlorofluorescin, that is detectable with a spectrofluorometer.

Oxidative stress was induced by incubating HepG2 cells with xanthine oxidase (XO), which is the important biological source of free radicals, and many references reveal cerebral microvascular injury resulting from the XO production of superoxide free radicals [27,28,29,30]. HepG2 cells were incubating with XO in the presence of its substrate xanthine (X) for a period of 3 h, and the oxidation of non-fluorescent DCFH-DA to the highly fluorescent DCF was used for the quantitation of intracellular ROS production. For testing the cytoprotective effects, HepG2 cells were pre-treated for 24 h with the tested compounds and then treated with XO and its substrate xanthine for periods of 3 h. As shown in Figure 2B, all dimers were able to slightly reduce the basal endogenous levels of ROS, and they were also able to scavenge most of the free radicals generated by the X/XO system, showing a higher activity than that of silibinin.

### 2.4. Serum Stability

**Figure 3:**
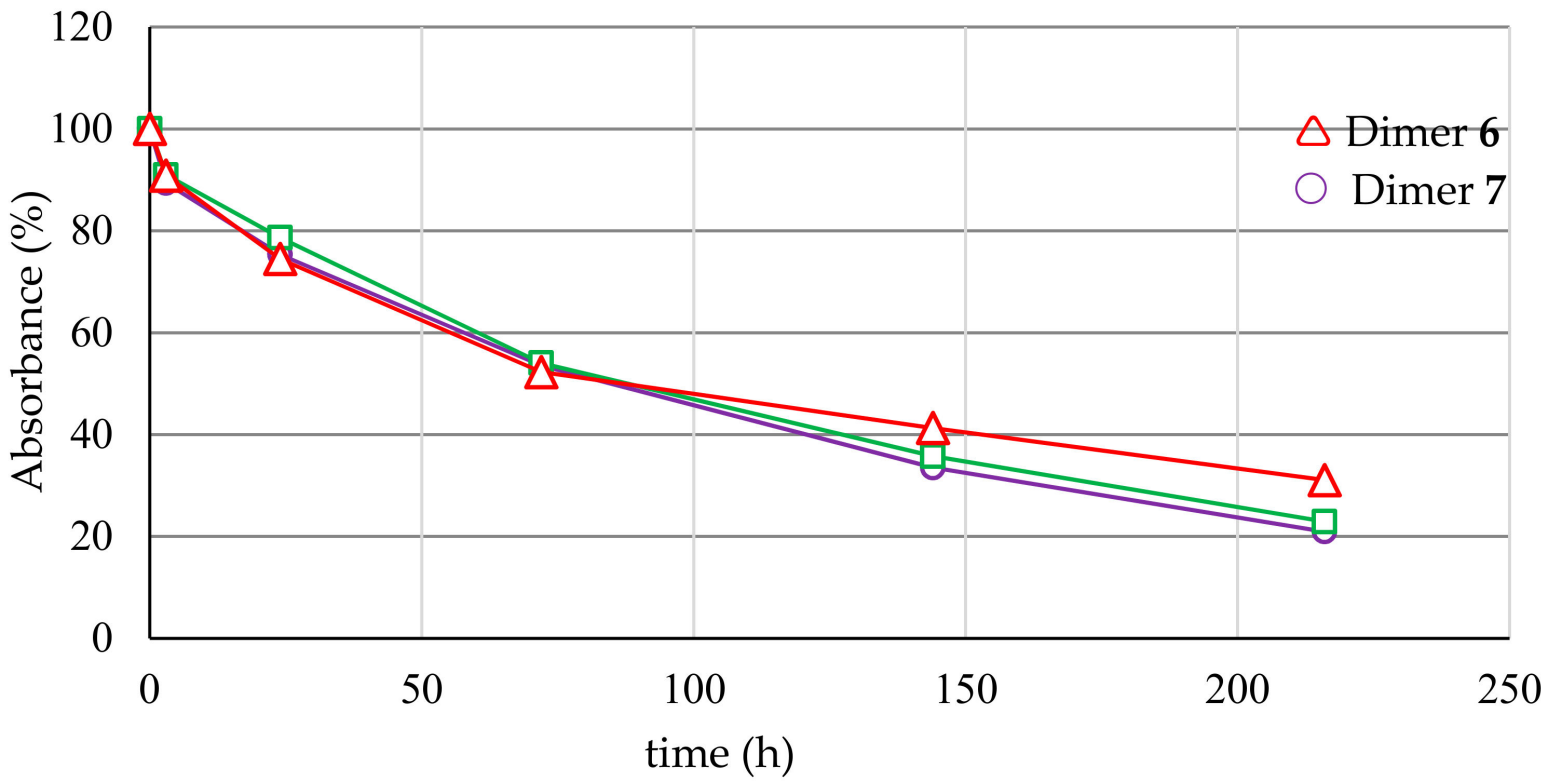
Abundance (%) of the dimers **6**–**8** treated with human serum at 37 °C and analysed by RP-HPLC (see Section 3.13).

Blood and extracellular fluid contain a large number of hydrolytic enzymes, such as cholinesterases, aldolases, lipases, dehydropeptidases, and alkaline phosphatases. Several functional groups, such as ester, amide, carbamate, lactam, lactone, sulphonamide and, of course, phosphate, are susceptible to plasma degradation. If the new compound has affinity for one of these enzymes and has a hydrolysable group in the right position, then it can be decomposed in the plasma. Hydrolysis in plasma can be a major cause of compound clearance, and pharmacologically efficacious concentrations may not be achievable in vivo in a pre-clinical study. With the presence of a phosphodiester bridge, which could be susceptible to the action of endogenous phosphatase before the active complex can reach its molecular target, the stability of the new synthesized conjugates was evaluated in human serum and was monitored over time by HPLC analysis (Figure 3).

As shown in Figure 3, the abundance of the dimers decreases slowly, recording half-lives of 81–87 h (Table 1).

### 2.5. Water Solubility

As reported in the literature, the water solubility of silibinin changes according to the experimental conditions, such as pH and temperature [8,31]. In this section, we determined the solubility of the new dimers **6**–**8** and silibinin (**1**) in water.

**Figure 4:**
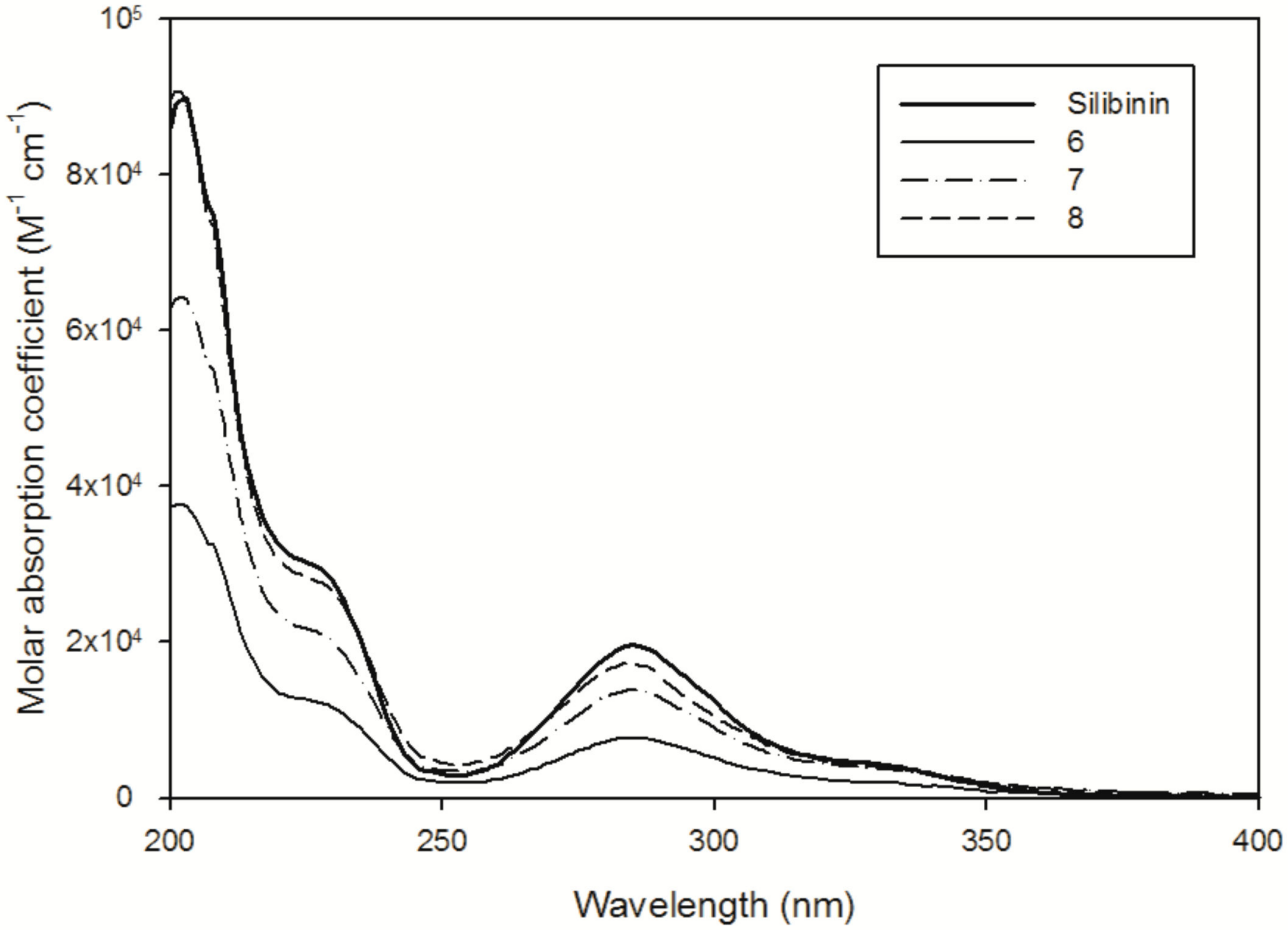
UV-Vis spectra of the silibinin in H_2_O-CH_3_CN (98:2) solution and the dimers in H_2_O at pH 6.5.

Solubility experiments were performed by dissolving the selected compound in Milli-Q H_2_O for 10 min under magnetic stirring in the dark. Molar absorption coefficients (*ε*) for each compound in water were determined from calibration curves (absorbance vs. concentration), with successive dilutions of a stock solution using the Beer-Lamberts law. For silibinin, the stock solution was prepared in CH_3_CN (1.18 mg in 1 mL), and absorption spectra were recorded by diluting the silibinin solution in H_2_O (2% of CH_3_CN used to increase the solubility and, as a consequence, quantification limit). UV-vis spectra are presented in Figure 4, revealing the presence of an absorption band that is centred at approximately 288 nm, corresponding to the π–π* transition, and at approximately 334 nm, which could be attributed to the transition n–π* that occurs on the oxygen of ketones groups. The silibinin was only slightly dissolved when 0.5 mg was suspended in 10 mL of H_2_O, and after filtration, the absorption spectrum revealed a maximum at 288 nm with an absorption of ~0.018. The saturated concentration of silibinin in H_2_O was estimated to be 0.9 µM, corresponding to ~0.44 mg·L^−1^ (Table 2). The solution was also kept in the dark and stirred for 12 h without any significant solubility increase.

To investigate the possible increased solubility at a higher pH, a phosphate buffer solution at pH 8.0 was prepared (buffer concentration of 1 mM), and 0.35 mg of silibinin was suspended in 15 mL. After 10 min of magnetic stirring followed by filtration through a 0.45-µm filter, the UV-vis spectrum revealed the presence of two absorption maxima: the first at 288 nm, attributed to the silibinin with an absorption of ~0.02 (in agreement with solubility determination at pH 6.5), and the second centred at approximately 330 nm, indicating the formation of silibinin degradation products in the alkaline solution. The solubility experiments that are summarized in Table 2 indicate that the dimers were completely dissolved (0.1 mg in 10 mL) in H_2_O, and solubility can be estimated to be ≥19.5 µM, corresponding to >20.3 mg·L^−1^.

### 2.6. Reactivity with *^1^*O*_2_* and HO∙

The reactivity of the silibinin and the dimers with O21 and ${\mathrm{HO}}^{•}$ is determined using Rose Bengal (RB) and hydrogen peroxide (H_2_O_2_) as respective ROS sources. For singlet oxygen, the steady-state concentration in the solution [O21]_SS_ in H_2_O was determined by following tryptophan disappearance at different initial concentrations. The kinetic approach is summarized in reactions (R1)–(R3):

(R1) RB→O2, hνO21      RO21f(Ms−1)

(R2) O21→O23       k1O2(s−1)

(R3) O21+ TRP→products  kO21,TRP(M−1s−1)

where RO21fbeing the rate of formation of O21 upon irradiation of RB, $k_{{}^{1}O_{2}}$ is the pseudo-first decay of singlet oxygen in H_2_O (2.4 × 10^5^ s^−1^), and $k_{{}^{1}O_{2},TRP}$ is the second order rate constant between ${}^{1}O_{2}$ and TRP (3.2 × 10^7^ M^−1^·s^−1^). Application of the steady-state approximation to ${}^{1}O_{2}$ leads to the following equation:

(EQ1) $$\frac{1}{R_{TRP}^{d}}=\frac{1}{R_{{}^{1}O_{2}}^{f}}(1+\frac{k_{{}^{1}O_{2}}}{k_{{}^{1}O_{2},TRP}[TRP]})$$

$\frac{1}{R_{TRP}^{d}}$ data vs. different TRP concentrations can be fitted with a linear equation, y=ax+b, leading to a quantification of the singlet oxygen steady state concentration ([${}^{1}O_{2}$]_SS_) that is equal to the ratio $R_{1}{}_{O_{2}}^{f}/k_{{}^{1}O_{2}}$. The dimer reactivity towards singlet oxygen was similar to the value determined for silibinin (see dimer **6** or approximately 35% lower in the case of dimer **8**—Table 3). The second order rate constant was on the same order of magnitude as that reported in the literature for molecules with a similar structure. Morales and co-workers [32] reported a reactivity ranging from 2.4 to 13.4 × 10^7^ M^−1^·s^−1^ for flavonoid derivatives such as quercetin and morin.

**Figure 5:**
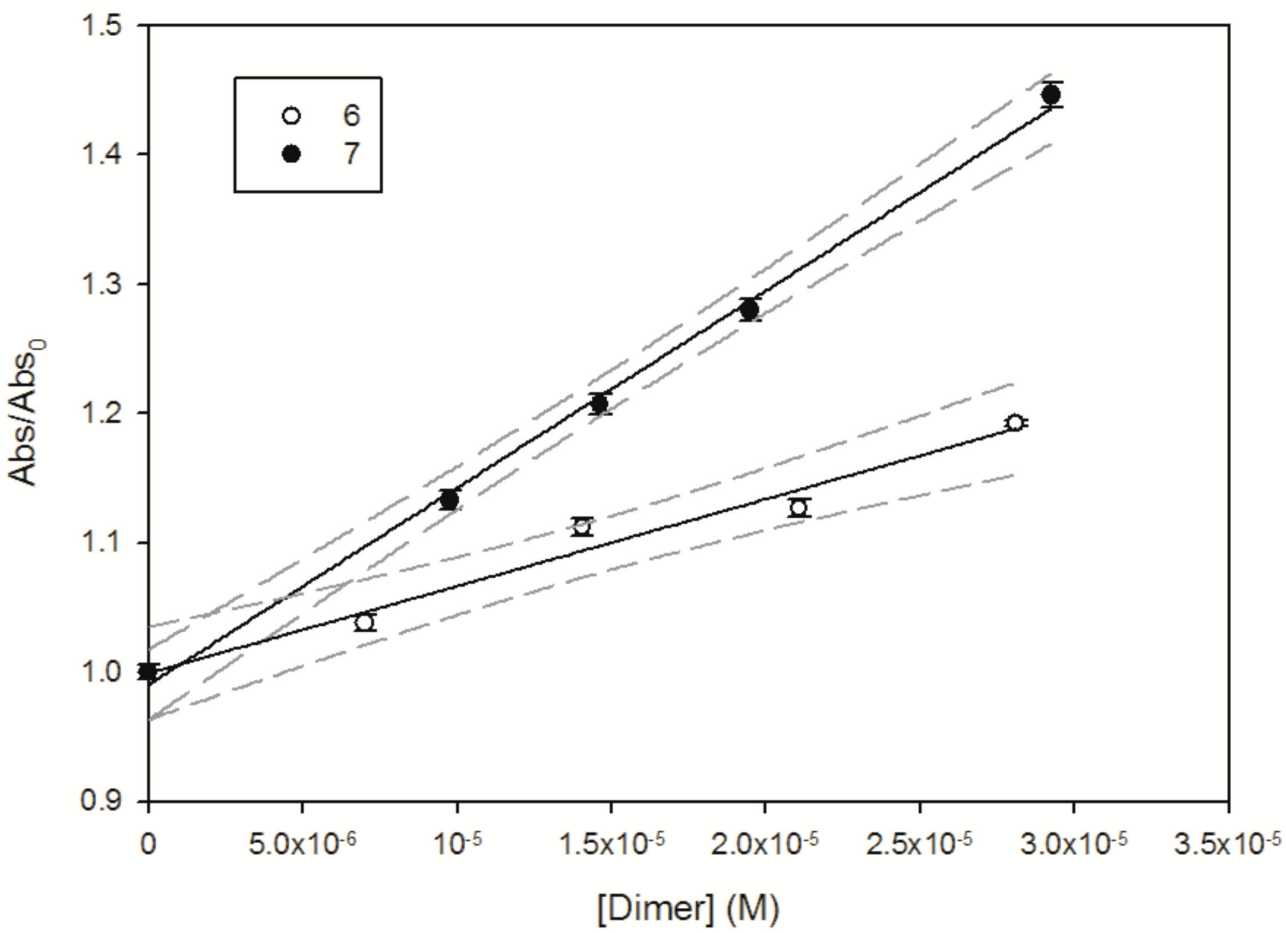
Abs/Abs_0_ measured at 475 nm as a function of **6** (PLSd 3-3) and **7** (PLSd 3-9′′). The solid line represents the linear fit of the experimental data, and dashed lines denote the 95% confidence interval of this fit.

Experimental data such as those reported in Figure 5 for dimers **6** and **7** (Abs/Abs_0_ followed at 475 nm vs. dimer concentration) are fitted with a linear Equation (EQ1), leading to the estimation of the second order rate constant between the hydroxyl radical and the selected molecule.

Estimation of the second order rate constant indicates that the most reactive species are dimers **7** and **8**, with a second order rate constant ≥ 1.5 × 10^10^ M^−1^·s^−1^ (see Table 4 summarizing the experimental results). Wang and co-workers [33] investigated the reactivity of the hydroxyl radical with phenolic compounds to estimate their antioxidative ability using aqueous pulse radiolysis. A value of 1.5 × 10^10^ M^−1^·s^−1^ was found for quercetin, which is closest to the value estimated for green tea polyphenols. Interestingly, Husain et al. [34] reported that the reactivity of flavonoids towards photogenerated hydroxyl radical increases with the number of hydroxyl groups in the aromatic ring.

## 3. Materials and Methods

### 3.1. General Methods

Reactions for the synthesis of dimers were monitored by thin layer chromatography (TLC) on precoated silica gel plates F254 (Merck, Darmstadt, Germany) and column chromatography was performed on Merck Kieselgel 60 (70–230 mesh). For the ESI MS analyses, a Micromass ZQ Instrument (Waters, Milan, Italy) equipped with an electrospray source was used. MALDI TOF mass spectrometric analyses were performed on a Voyager-De Pro MALDI mass spectrometer (PerSeptive Biosystems, Framingham, MA, USA). Absorption spectra were measured with a Cary 100 Scan UV-Vis spectrophotometer (Varian, Santa Clara, CA, USA) using a 1-cm path length quartz cell.

### 3.2. Chemicals

Silibinin (purity ≥98% by HPLC) was purchased from Sigma-Aldrich (Milan, Italy). HPLC grade CH_3_CN and CH_3_OH were purchased from Carlo Erba Reagents (Milan, Italy) and Sigma-Aldrich. Hydrogen peroxide (H_2_O_2_) (30%) was purchased from Fluka (Milan, Italy). Unless otherwise indicated, all other chemicals were obtained from Sigma Aldrich, and their purity was >98%. Fresh aqueous solutions of hydrogen peroxide, Rose Bengal and tryptophan were prepared prior to each experiment. The concentration of the stock solution of H_2_O_2_ in Milli-Q H_2_O (for the laser flash photolysis experiments) was determined using a molar absorption coefficient of 38.1 ± 1.4 M^−1^·cm^−1^ at 240 nm [20].

### 3.3. Synthesis of Silibinin Building Block ***2***

Silibinin (**1**, 1.5 g, 3.1 mmol) previously dried by repeated co-evaporation with anhydrous THF was dissolved in anhydrous THF (8 mL) and TEA (2.1 mL, 15.4 mmol). Isobutyryl chloride (0.980 mL, 9.3 mmol) was added, and the mixture was kept at 0 °C for 30 min. After reaction completion as monitored by TLC, the reaction was stopped by the addition of CH_3_OH, and the mixture was extracted with CH_2_Cl_2_. The organic phase was dried over anhydrous Na_2_SO_4_ and concentrated under reduced pressure, and the crude material was purified by column chromatography (CHCl_3_-acetone, 96:4, *v*/*v*). The derivative 3,5,7,4′′-tetra-*O*-isobutyrylsilibinin (**2**) was obtained as a pale amorphous solid (0.365 g, 0.47 mmol, 15%). R*_f_* = 0.6 (benzene/EtOAc, 80:20, *v*/*v*). ^1^H-NMR (CDCl_3_, 400 MHz, room temperature, mixture of two diastereoisomers): δ = 7.15–6.96 (6H, complex signals, H-2′, H-5′, H-6′, H-2′′, H-5′′, H-6′′), 6.77 (1H, complex signal, H-8), 6.58 (1H, s, H-6), 5.71 and 5.69 (1H, d, *J* = 12.2/12.3 Hz, H-3), 5.38 (1H, m, H-2), 5.08 and 5.06 (1H, d, *J* = 8.3/8.7 Hz, H-7′′), 4.04 (1H, m, H-8′′), 3.88–3.83 (4H, overlapped signals, OCH_3_, H-9′′a), 3.58 (1H, dd, *J* = 3.2 Hz, *J* = 12.0 Hz, H-9′′b), 2.98–2.74 (3H, complex signals, CH of isobutyryl groups), 2.62–2.52 (1H, m, CH of isobutyryl group), 1.41–1.25 (18H, m, CH_3_ of isobutyryl groups), 1.15–1.10 (6H, m, CH_3_ of isobutyryl group) ppm. ^13^C-NMR (CDCl_3_, 100 MHz, room temperature, mixture of diastereoisomers): δ = 185.0, 175.2, 175.1, 175.0, 174.1, 162.5, 162.4, 156.5, 151.7, 151.6, 144.0, 144.1, 143.7, 140.6, 134.5, 128.7, 123.1, 123.0, 121.0, 120.8, 119.8, 119.7, 117.1, 117.0, 116.6, 116.4, 111.2, 111.1, 108.7, 81.1, 81.0, 78.3, 78.4, 76.0, 75.9, 72.9, 72.8, 61.5, 56.1, 56.0, 34.3, 34.0, 33.7, 19.0, 18.8, 18.7, 18.6 ppm. ESI-MS (positive ions): *m*/*z* calculated for C_41_H_46_O_14_ = 762.29; found: 763.84 [MH]^+^.

### 3.4. Synthesis of Silibinin Building Block ***3***

Silibinin (**1**, 1.0 g, 2.7 mmol) previously dried by repeated co-evaporation with anhydrous THF was dissolved in anhydrous THF (8 mL) and TEA (2.1 mL, 15.4 mmol), and isobutyryl chloride (1.1 mL, 10.4 mmol) was added. The mixture was kept at 0 °C for 30 min. After it was monitored by TLC, the reaction was stopped by the addition of CH_3_OH, and the mixture was extracted with CH_2_Cl_2_; the organic phase was then dried over anhydrous Na_2_SO_4_ and concentrated under reduced pressure. The crude material was purified by column chromatography (CHCl_3_-acetone, 96:4, *v*/*v*). The derivative 5,7,4′′,9′′-tetra-*O*-isobutyrylsilibinin (**3**) was obtained as a pale amorphous solid (0.447 g, 0.58 mmol, 28%). R*_f_* = 0.9 (benzene/EtOAc, 80:20, *v*/*v*). ^1^H-NMR (CDCl_3_, 400 MHz, room temperature, mixture of two diastereoisomers): δ = 7.21–6.98 (complex signals, 6H, H-2′, H-5′, H-6′, H-2′′, H-5′′, H-6′′), 6.76 (1H, complex signal, H-8), 6.59 (1H, s, H-6), 5.07 (1H, d, *J* = 12.8 Hz, H-2), 5.00 (1H, d, *J* = 8.3 Hz, H-7′′), 4.56 and 4.55 (1H, d, *J* = 12.3/12.4 Hz, H-3), 4.44 (1H, dd, *J* = 2.3 Hz, *J* = 12.9 Hz, H-9′′a), 4.26 (1H, m, H-8′′), 3.98 (1H, dd, *J* = 2.3 Hz, *J* = 12.8 Hz, H-9′′b), 3.86 (3H, s, OCH_3_), 2.98–2.76 (3H, complex signals, CH of isobutyryl groups), 2.61 (1H, ept, *J* = 6.8 Hz, CH of isobutyryl group), 1.36 (18H, complex signals, CH_3_ of isobutyryl groups), 1.21 (6H, m, CH_3_ of isobutyryl group) ppm. ^13^C-NMR (CDCl_3_, 100 MHz, room temperature, mixture of two diastereoisomers): δ = 191.2, 176.4, 175.0, 174.9, 174.1, 163.1, 157.0, 151.7, 151.5, 144.0, 143.5, 140.8, 134.3, 129.3, 123.2, 121.4, 121.2, 119.8, 117.5, 117.4, 116.6, 116.5, 111.1, 110.9, 109.7, 108.9, 83.2, 76.2, 75.9, 75.8, 73.1, 73.0, 62.4, 56.0, 34.3, 34.1, 34.0, 33.9, 19.0, 18.9, 18.7, 18.6 ppm. ESI-MS (positive ions): *m*/*z* calculated for C_41_H_46_O_14_ = 762.29; found: 763.66 [MH]^+^.

### 3.5. Synthesis of Silibinin Building Block ***4***

DIEA (390 μL, 2.22 mmol) and 2-cyanoethyl-*N*,*N*-diisopropylaminochlorophosphoramidite (107 μL, 0.48 mmol) were added to 3,5,7,4′′-tetra-*O*-isobutyrylsilibinin (**2**, 240.0 mg, 0.33 mmol) dissolved in anhydrous CH_2_Cl_2_ (5 mL). After 30 min, the solution was diluted with EtOAc, and the organic phase was washed twice with brine and then concentrated. Silica gel chromatography of the residue (*n*-hexane-EtOAc, 30:70, *v*/*v*, in the presence of 1% of Et_3_N) afforded the desired compound **4** (205.0 mg, 0.24 mmol) with a 65% yield. R*_f_* = 0.8 (*n*-hexane/EtOAc, 60:40, *v*/*v*). ^1^H-NMR (CDCl_3_, 500 MHz, room temperature, mixture of four diastereoisomers): δ = 7.11–6.94 (6H, overlapped signals, H-2′, H-3′, H-6′, H-2′′, H-5′′, H-6′′), 6.76 (1H, complex signals, H-8), 6.56 (1H, s, H-6), 5.69 (×2), 5.68 and 5.67 (1H, d, *J* = 12.2 Hz, H-3), 5.38, 5.37 (×2) and 5.36 (1H, d, *J* = 12.2 Hz, H-2); 5.03 (×2) and 5.01 (×2) (1H, d, *J* = 7.2/7.5 Hz, H-7′′), 4.00–3.51 (12H, complex signals, OCH_3_, H-8′′, H-9′′a, H-9′′b, OCH_2_CH_2_CN, N[CH(CH_3_)_2_]_2_ OCH_2_CH_2_CN), 3.01–2.68 (3H, m, CH of isobutyryl groups), 2.56 (1H, m, CH of isobutyryl group) 1.36–0.95 (36H, complex signals, CH_3_ of isobutyryl groups, N[CH(CH_3_)_2_]_2_) ppm. ^13^C-NMR (CDCl_3_, 100 MHz, room temperature, mixture of diastereoisomers): δ = 185.0, 175.2, 175.1, 175.0, 174.1, 162.5, 162.4, 156.5, 151.7, 151.5, 144.5, 143.4, 143.3, 140.5, 134.7, 128.3, 128.2, 123.0, 122.9, 121.0, 120.9, 120.8, 119.9, 119.8, 119.7, 117.7, 117.5, 117.1, 116.4, 116.3, 111.5, 111.4, 111.3, 111.1, 111.0, 110.8, 108.7, 81.2, 81.1, 76.2, 75.9, 75.8, 72.9, 72.8, 62.7, 62.5, 62.2, 62.0, 58.8, 58.6, 58.5, 58.3, 56.0, 55.9, 53.4, 43.3, 43.2, 34.2, 34.0, 33.9, 33.6, 24.6, 24.5, 19.0, 18.8, 18.7, 18.6 ppm. ^31^P-NMR (CDCl_3_, 161.98 MHz): δ = 150.2, 149.8, 149.7 ppm. ESI-MS (positive ions): *m*/*z* calculated for C_50_H_63_N_2_O_15_P = 962.40; found: 963.92 [MH]^+^.

### 3.6. Synthesis of Silibinin Building Block ***5***

DIEA (390 μL, 2.22 mmol), and 2-cyanoethyl-*N*,*N*-diisopropylaminochlorophosphoramidite (107 μL, 0.48 mmol) were to 5,7,4′′,9′′-tetra-*O*-isobutyrylsilibinin (**3**, 240.0 mg, 0.33 mmol) dissolved in anhydrous CH_2_Cl_2_ (5 mL). After 1 h, the solution was diluted with EtOAc, and the organic phase was washed twice with brine and then concentrated. Silica gel chromatography of the residue (*n*-hexane-EtOAc, 3:7, *v*/*v*, in the presence of 1% of Et_3_N) afforded the desired compound **5** (205.0 mg, 0.24 mmol) in a 65% yield. R*_f_* = 0.8 (*n*-hexane/EtOAc, 60:40, *v*/*v*). ^1^H-NMR (CDCl_3_, 500 MHz, room temperature, mixture of four diastereoisomers): δ = 7.30–6.97 (6H, overlapped signals, H-2′, H-3′, H-6′, H-2′′, H-5′′, H-6′′), 6.73 (1H, complex signals, H-8), 6.54 (1H, complex signal, H-6), 5.02 (×2) and 5.01 (×2) (1H, d, *J* = 7.8/8.0 Hz, H-7′′), 4.94 (1H, d, *J* = 9.3 Hz, H-2), 4.43 (1H, complex signal, H-3), 4.34 (complex signal, 1H, H-9′′a), 4.20 (1H, complex signal, H-8′′), 4.10–3.94 (1H, complex signals, H-9′′b), 3.86 (3H, complex signal, OCH_3_), 3.54–3.34 (6H, complex signals, OCH_2_CH_2_CN, N[CH(CH_3_)_2_]_2_ OCH_2_CH_2_CN) 2.96–2.74 (3H, complex signals, CH of isobutyryl groups), 2.65 (1H, complex signal, CH of isobutyryl group), 1.40–1.10 (36H, complex signals, CH_3_ of isobutyryl groups, N[CH(CH_3_)_2_]_2_) ppm. ^13^C-NMR (CDCl_3_, 100 MHz, room temperature, mixture of diastereoisomers): δ = 189.0, 188.3, 176.4, 176.3, 174.9, 174.2, 174.1, 162.3, 156.3, 156.1, 149.9, 143.9, 143.8, 143.4, 143.3, 140.8, 135.9, 134.2, 129.5, 129.4, 129.3, 123.2, 121.7, 121.6, 119.7, 119.6, 117.8, 117.5, 117, 4, 117.3, 116.8, 116.6, 111.3, 111.1, 111.0, 108.6, 83.1, 82.9, 76.2, 75.8, 75.6, 75.2, 62.4, 62.3, 59.7, 59.5, 58.5, 58.3, 56.0, 43.5, 43.4, 34.2, 34.1, 33.9, 24.8, 24.7, 24.6, 24.2, 24.1, 19.0, 18.9, 18.8, 18.7, 18.6 ppm. ^31^P-NMR (CDCl_3_, 161.98 MHz): δ = 153.2, 153.0, 152.3, 152.2 ppm. ESI-MS (positive ions): *m*/*z* calculated for C_50_H_63_N_2_O_15_P = 962.40; found: 963.43 [MH]^+^.

### 3.7. Synthesis of Phosphate-Linked Silibinin Dimers ***6**–**8***

The coupling reaction between the phosphoramidites **4** or **5** and the suitable silibinin building block **2** or **3** was carried out by suspending 240 mg (0.32 mmol) of derivative **2** (or **3**) in a solution of 4,5-dicyanoimidazole in acetonitrile (DCI, 0.45 M) and by adding 3-Å molecular sieves. After a few min, the mixture was added to 270 mg (0.28 mmol) of silibinin phosphoramidite **4** (or **5**) and stirred for 1 h. Upon completion of reaction as monitoed by TLC analysis, *tert*-butyl hydroperoxide (TBHP) solution in decane (150 μL, 5.5 M) was added, and after 30 min, the solvent was removed under vacuum, and the crude material was purified by column chromatography (eluent CHCl_3_/CH_3_OH, 99:1 to 95:5, *v*/*v*). The material obtained was subsequently subjected to treatment with a 28% NH_4_OH aqueous solution and methanol (1:1, *v*/*v*) at 50 °C for 3 h for the full deprotection.

Compound **6** (pale yellow, 49 mg, 17%): ^31^P-NMR (161.98 MHz, DMSO-*d*_6_, room temperature, mixture of diastereoisomers) δ = −1.61. MS (MALDI-TOF) *m*/*z* calcd. for C_50_H_43_O_22_P = 1026.20, found 1025.12 [M − H]^−^, 1047.09 [M + Na − 2H]^−^.

Compound **7** (pale yellow, 43 mg, 15%): ^31^P-NMR (161.98 MHz, DMSO-*d*_6_, room temperature, mixture of diastereoisomers) δ = −1.64. MS (MALDI-TOF) *m/z* calcd. for C_50_H_43_O_22_P = 1026.20, found 1025.28 [M − H]^−^, 1047.09 [M + Na − 2H]^−^.

Compound **8** (pale yellow, 58 mg, 20%): ^31^P-NMR (161.98 MHz, DMSO-*d*_6_, room temperature, mixture of diastereoisomers) δ = −1.62. MS (MALDI-TOF) *m*/*z* calcd. for C_50_H_43_O_22_P = 1026.20, found 1025.11 [M − H]^−^, 1048.99 [M + Na − 2H]^−^.

### 3.8. HPLC Analysis and Purification of ***6**–**8***

The sample solutions were prepared by dissolving and sonicating the accurately weighed compound (**6**–**8**) in H_2_O/CH_3_OH (1:1, *v*/*v*). The obtained solution was filtered using nylon filters (pore size = 0.45 µm) and then purified. The HPLC analysis was performed with an LC-9A HPLC system (Shimadzu, Milan, Italy) equipped with a Shimadzu SPD-6A detector using a RP18 column (LUNA, Phenomenex, Bologna, Italy, 5 μm particle size, 10.0 mm × 250 mm i.d.) eluted with ammonium acetate 0.1 M (pH = 7.0) with a linear gradient of 5–100% ACN for 20 min (flow = 0.8 mL·min^−1^) and monitored at 288 nm. The preparative HPLC method was performed with a Shimadzu LC-8A PLC system (Shimadzu, Milan, Italy) equipped with a Shimadzu SCL-10A VP System control and Shimadzu SPD-10A VP UV-VIS Detector. A Phenomenex Gemini C18-110A preparative column (5-μm particle size, 250 mm × 21.2 mm i.d.) was eluted with ammonium acetate 0.1 M with a linear gradient of 20–100% ACN for 50 min (flow = 7 mL/min). The chromatograms were monitored at 288 and 254 nm. The HPLC system was controlled by the Shimadzu LC Real Time Analysis software (LCsolution Version 1.25, Shimadzu, Milan, Italy). The recovered material was desalted on a Chromafix^®^ C18 column (Macherey Nagel, Milan, Italy) eluted with H_2_O/CH_3_OH (8:2, *v*/*v*) and converted into the corresponding sodium salt by cation exchange on a DOWEX resin (Na^+^) column, affording compounds **6**–**8** in good yields (17%, 15% and 20%, respectively).

### 3.9. DPPH Test

The free radical scavenging activities of the test compounds at different concentrations were evaluated by their abilities to quench the stable 1,1-diphenyl-2-picrylhydrazyl radical (DPPH) in vitro. A DPPH solution (20 mg/mL) was prepared in methanol. The compounds were dissolved in methanol to prepare the stock solution (1 mM). A freshly prepared DPPH solution was aliquoted into test tubes, and analogue solutions (1 mM^−1^ μM) were added to every test tube so that the final volume was 2.25 mL. After 10 min, the absorbance was read at 515 nm. Silibinin and quercetin were used as reference standards and dissolved in methanol to make a stock solution at the same concentration (1 mM).

### 3.10. Evaluation of Cell Viability (MTT Assay)

Cell viability was measured using an MTT [3-(4,5-dimethylthiazol-2-yl)-2,5-diphenyl tetrazolium bromide] assay on HepG2 cells. Cells were plated at a density of 2 × 10^4^ cells·well^−1^ in 96-well plates, and after 36 h, the medium was replaced with fresh medium supplemented with the tested compounds. After 24 h, the cell medium was replaced with 200 μL of MTT solution (5 mg·mL^−1^ stock solution in PBS) and diluted with culture medium to a final concentration of 0.5 mg·mL^−1^. After a 4 h incubation at 37 °C, this solution was removed, and the formazan was solubilized in 100 μL of DMSO. The absorbance was monitored at 572 nm using an automated microplate reader (PowerWave XS2; BioTek Instruments Inc., Winooski, VT, USA). Cell viabilities were calculated by comparing the results to those for control cells considered 100% viable. The cells were assayed in triplicate independent experiments.

### 3.11. Xanthine/Xanthine Oxidase (X–XO) Assay

Cells (2 × 10^4^ cells) were seeded on 96-well plates. The cells were pretreated with each dimer (**6**–**8**) at concentrations of 20 μM for 24 h and then exposed to X (0.5 mM) and XO (5 mU·mL^−1^) for 3 h. The cell plates were washed twice with Hanks’ Balanced Salt Solution (HBSS) and incubated in the same buffer containing 10 μM DCFH-DA for 1 h at 37 °C in the dark. Oxidation of the dye by intra-cellular ROS generates a fluorescent 2′,7′-dichlorofluorescin (DCF) signal. Intracellular fluorescence was detected using a SPECTRA max GEMINI spectrofluorometer (Molecular Devices, Rome, Italy, excitation wavelength, 495 nm; emission wavelength, 530 nm).

### 3.12. Statistical Analysis

Data were collected and are expressed as the mean ± standard deviation of three independent experiments. Statistical analysis was performed by ANOVA tests. A *p* < 0.05 was considered significant.

### 3.13. Serum Stability

Tests were performed in 500 μL of 50 mM Tris∙HCl (pH 8.0 containing 10 mM MgCl_2_), 50 μL of 10 μM solution of **6**–**8**, and human serum from human male AB plasma (400 μL). All reactions were incubated at 37 °C and small aliquots of the mixture were taken at defined time intervals, kept at 85 °C for 5 min, equilibrated at rt, and analysed by RP-HPLC. HPLC analyses were carried out by RP-HPLC using a Phenomenex Luna C18 column (5 μm particle size, 10.0 mm × 250 mm i.d., Phenomenex, Bologna, Italy) eluted with 0.1 M ammonium acetate with a linear gradient 20–100% ACN for 30 min (flow = 1.5 mL·min^−1^).

### 3.14. Singlet Oxygen Reactivity

**Figure 6:**
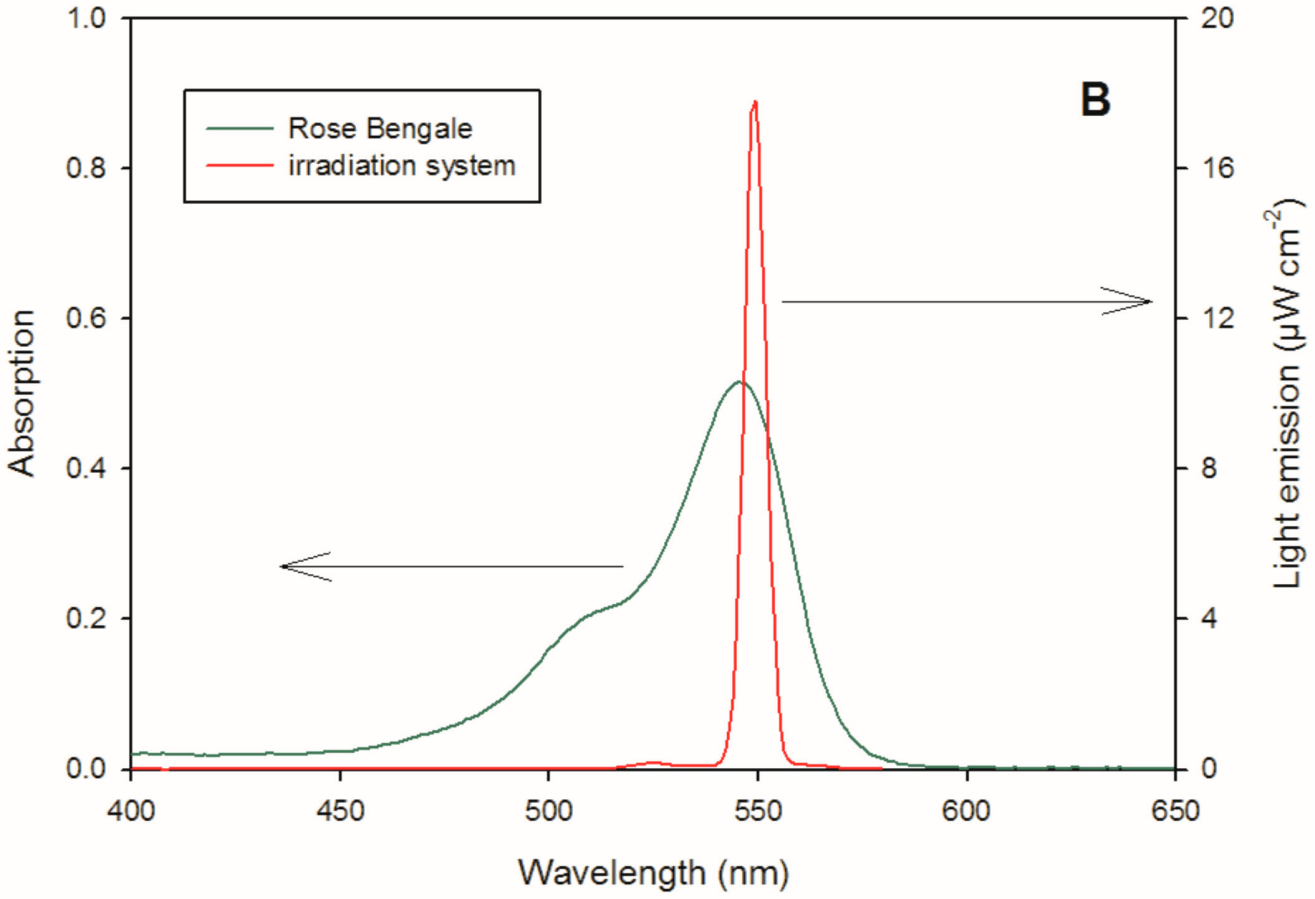
Emission spectrum reaching the solution (1000 W xenon lamp equipped with a monochromator) and absorption spectrum of a 10 µM RB solution.

To estimate the reactivity of silibinin and the dimers towards singlet oxygen, solutions were irradiated in a quartz cell of 1 cm path length using 545 nm centred irradiation (1000 W xenon lamp equipped with a monochromator) in the presence of 100 µM RB as a photosensitizer for singlet oxygen generation [35]. An aliquot of the solution was collected at fixed intervals of time and analysed by liquid chromatography. Figure 6 shows the absorption spectra of RB and the overlap with the emission spectra reaching the solution, recorded using fibre optics coupled with a CCD spectrophotometer (USD 2000, UV-vis 200–850 nm, Ocean Optics, Paris, France). A reference lamp (DH-2000-CAL, Ocean Optics) was used for calibration.

Silibinin was solubilized in THF, whereas the dimers were solubilized in an H_2_O/CH_3_CN (9/1, *v*/*v*) mixture. THF and CH_3_CN were used as solvents to increase the silibinin and dimer solubility and due to their similar reactivity with singlet oxygen compared to the water (i.e., only limited singlet oxygen scavenging is accounted for by the solvent) [36]. The degradation of silibinin and the dimer as determined as follows: an aliquot (200 µL) was taken at fixed interval times during irradiation, stored in the dark and monitored by ultra-high performance liquid chromatography using a Waters Acquity UPLC instrument equipped with a diode array detector and a Waters 2695 separation module. The gradient is as follows: a linear increase from 1% CH_3_CN and 99% H_2_O acidified with 0.1% of formic acid to 50% CH_3_CN within 5 min. Then, CH_3_CN was increased linearly to 80% for 2 min and maintained constant for 2 min. The column was a Water Acquity UPLC C18 (100 mm × 2.1 mm × 1.7 µm) with a flow rate of 0.3 mL·min^‒1^. The retention times of silibinin and the 3-3, 3-9′′ and 9′′-9′′ dimers were 5.4, 6.3, 7.0 and 7.3 min, respectively, with the detection wavelength set at 288 nm.

The time evolution of silibinin and dimers in the presence of singlet oxygen could be fitted with a pseudo-first order equation $C_{t}=C_{0}\exp^{-k_{{}^{1}O_{2}}t}$ where *C*_0_ is the initial concentration of compound, *C_t_* the concentration at time *t* and $k_{{}^{1}O_{2}}$ the pseudo-first order degradation rate constant of target compound (X). The degradation rate ($R_{X}^{d}$) was determined as $R_{X}^{d}=k_{{}^{1}O_{2}}C_{0}$.

Singlet oxygen steady-state concentration [${}^{1}O_{2}$]_SS_ using 100 µM RB under irradiation is quantified under experimental conditions using tryptophan as probe. The transformation rate of silibinin or dimers in the presence of singlet oxygen is given by the following relation: $R_{X}^{d}=k_{{}^{1}O_{2},X}^{II}[{}^{1}O_{2}{]}_{\mathrm{SS}}\left[X\right]$ where [X] is the concentration of silibinin or dimers in solution. The second order rate constant between target molecule and singlet oxygen ($k_{{}^{1}O_{2},X}^{II}$(M^−1^·s^−1^)) can be determined as: $k_{{}^{1}O_{2},X}^{II}=\frac{R_{X}^{d}}{{\left[{}^{1}O_{2}\right]}_{\mathrm{SS}}[X]}$. Singlet oxygen steady-state concentration [${}^{1}O_{2}$]_SS_ is quantified with tryptophan as probe using experimental conditions adopted for silibinin and dimmers considering a second order rate constant between TRP and ${}^{1}O_{2}$ of 3.2 × 10^7^ M^−1^·s^−1^ [37].

### 3.15. Hydroxyl Radical ($HO^{•}$) Generation and Reactivity Estimation

Due to the high reactivity of THF with hydroxyl radical (~4.0 × 10^9^ M^−1^·s^−1^) [38], stock solutions of H_2_O_2_, thiocyanate (${\mathrm{SCN}}^{-}$), silibinin and the dimers were prepared in Milli-Q H_2_O or CH_3_CN (for silibinin), and an appropriate volume was mixed just before each experiment to obtain the desired concentration. Experiments in pure H_2_O were performed for the dimers, whereas for silibinin, a CH_3_CN/H_2_O (9:1) solution was used. All experiments were performed at ambient temperature (295 ± 2 K) and in an aerated solution.

Hydroxyl radicals were generated from 266 nm excitation of the H_2_O_2_ solution, corresponding to the fourth harmonic of a GCR 130-01 Nd:YAG laser system instrument (Quanta Ray) used in a right-angle geometry with respect to the monitoring light beam. The single pulse was 9 ns in duration, and the energy was 40 mJ. Individual cuvette samples (~3 mL volume) were used for a maximum of two consecutive laser shots to avoid degradation product formation and interference. The transient absorbance at the pre-selected wavelength was followed by a detection system consisting of a pulsed xenon lamp (150 W), a monochromator and a photomultiplier (1P28). A spectrometer control unit was used to synchronise the pulsed light source and programmable shutters with the laser output. The signal from the photomultiplier was digitised by a programmable digital oscilloscope (HP54522A) and analysed using a 32 bit RISC-processor kinetic spectrometer workstation.

The UV-vis spectrum of ${\mathrm{HO}}^{•}$ presents a maximum that is centred at 240 nm, and direct quantification was difficult due to the low molar absorption coefficient (ε_240nm_) of ~600 M^−1^·cm^−1^ [39]. For this reason, a kinetic approach based on ${\mathrm{SCN}}^{-}$ competition reactivity is generally adopted to estimate the second order rate constant of target compounds with ${\mathrm{HO}}^{•}$ in solution based on the following equations:

(R4) $$H_{2}O_{2}\overset{LFP\;266nm}{\to }{2\mathrm{HO}}^{•}$$

(R5) $${\mathrm{HO}}^{•}+\;{\mathrm{SCN}}^{-}\to {\mathrm{HO}}^{-}{+\;\mathrm{SCN}}^{•}$$

(R6) $${\mathrm{HO}}^{•}+\;X\to \mathrm{products}$$

(R7) $${\mathrm{SCN}}^{•}{+\;\mathrm{SCN}}^{-}\to {\mathrm{SCN}}_{2}^{•-}$$

The first step (R4) was hydrogen peroxide photolysis using a laser pulse at 266 nm followed by competition reactivity of the generated ${\mathrm{HO}}^{•}$with ${\mathrm{SCN}}^{-}$ (R5) and silibinin (or dimers) (R6). In the presence of a high concentration of ${\mathrm{SCN}}^{-}$, thiocyanate radical (${\mathrm{SCN}}^{•}$) reacts with another equivalent of thiocyanate to form a di-thiocyanate radical anion (${\mathrm{SCN}}_{2}^{•-}$) (R7), which has a strong absorption molar coefficient at 475 nm (ε_475nm_ = 7600 M^−1^·cm^−1^) [40].

The absorption of ${\mathrm{SCN}}_{2}^{•-}$ in the presence of different concentrations of ${\mathrm{HO}}^{•}$ quenchers (X) allows for the determination of the second-order rate constant between hydroxyl radicals and silibinin or the dimers using the following equation:

(E1) $$\frac{Abs_{0}}{Abs}=1+\frac{k_{{\mathrm{HO}}^{•},X}^{II}[X]}{k_{{\mathrm{HO}}^{•},{\mathrm{SCN}}^{-}}^{II}[{\mathrm{SCN}}^{-}]}$$

where $Abs_{0}$ and Abs are the transient absorption of $SCN_{2}^{•-}$ at 475 nm, respectively, with and without quencher; $k_{{\mathrm{HO}}^{•},{\mathrm{SCN}}^{-}}^{II}$ is the second-order rate constant between ${\mathrm{HO}}^{•}$ and thiocyanate, which is 1.2 × 10^10^ M^−1^·s^−1^ [41]; [${\mathrm{SCN}}^{-}$] is the initial concentration of thiocyanate (0.1 or 0.5 mM in our study); [X] is the concentration of target compound; and $k_{{\mathrm{HO}}^{•},X}^{II}$ is the second order rate constant with the hydroxyl radical. $\frac{Abs_{0}}{Abs}$ values vs. the concentration of quencher ([SY]) can be fitted using a linear equation, y=ax+b, in which $y=\frac{Abs_{0}}{Abs}$, b=1, x=[X], and $a=\;\frac{k_{{\mathrm{HO}}^{•},X}^{II}}{k_{{\mathrm{HO}}^{•},{\mathrm{SCN}}^{-}}^{II}[{\mathrm{SCN}}^{-}]}$. The $k_{{\mathrm{HO}}^{•},X}^{II}$ value can be determined to be equal to $a\;\times \;k_{{\mathrm{HO}}^{•},{\mathrm{SCN}}^{-}}^{II}[{\mathrm{SCN}}^{-}]$. Stock solutions of H_2_O_2_, ${\mathrm{SCN}}^{-}$, silibinin and the dimers were prepared in Milli-Q H_2_O or CH_3_CN (for silibinin), and an appropriate volume was mixed just before each experiment to obtain the desired concentration of all the species. Experiments in pure H_2_O were performed for the dimers, whereas for silibinin, 10% CH_3_CN in a H_2_O solution was used. All experiments were performed at ambient temperature (295 ± 2 K) and in aerated solutions.

## 4. Conclusions

New promising synthetic metabolites **6**–**8** named Phosphate-Linked Silibinin dimers were synthesized in very good yields, using an efficient synthetic strategy exploiting the well-known phosphoramidite chemistry. Their antioxidant properties were evaluated by different approaches (DPPH test, X/XO system, ROS reactivity). Knowing the strong limitations of silibinin, mainly due to its poor bioavailability, the water solubility of the new derivatives was evaluated. The new dimers (PLSd) presented a good water solubility (more than 20 mg·L^−1^) under circumneutral pH values, whereas the silibinin was found to be poorly soluble (less than 0.4 mg·L^−1^). In a preliminary study the antioxidant activities of the PLSd were evaluated by DPPH free radical scavenging test showing a pronounced increase of the activity for the dimers in comparison to the silibinin. The PLSd showed a very low toxicity towards HepG2 cells (even for long exposure times) and a half-life time in human serum of approximately three days at 37 °C. The cytoprotective activity of the PLSd was evaluated on HepG2 cells using the DCF assay where the oxidative stress tests were carried out using the xanthine/xanthine oxidase system. All the dimers showed a strong ability to scavenge most of the free radicals generated by X/XO on HepG2 cells and in all cases more efficiently than silibinin. Moreover, the anti-oxidant activities of the new derivatives were further investigated towards two different ROS, ${}^{1}O_{2}$ and ${\mathrm{HO}}^{•}$, respectively produced by Rose Bengal and hydrogen peroxide sources. The ability to scavenge ${}^{1}O_{2}$ in H_2_O was tested and despite a similar reactivity for both dimers and silibinin, indeed a higher reactivity towards ${\mathrm{HO}}^{•}$ (about two times) was estimated for the dimers **7** and **8** respect to the silibinin. Therefore, the second order rate constant for the dimers **7** and **8** is major than 1.5 × 10^10^ M^−1^·s^−1^ value very similar to that reported for a known potent antioxidant as quercetin. The combination of the great antioxidant ability with low toxicity as well as their serum stability, makes the PLSd promising synthetic metabolites in the large class of polyphenols.

## Figures and Tables

**Table 1:** Characterization of the Phosphate-Linked Silibinin dimers **6**–**8**.

Compound	MALDI	*t_R_* (min) ^a^	DPPH IC_50_ (mM)	t_1/2_ (h)
**6**	Calcd. for C_50_H_43_O_22_P = 1026.20 found [M − H]^−^ = 1025.12; [MNa − H]^−^ = 1047.09	13.6	0.58 ± 0.04	86.7
**7**	Calcd. for C_50_H_43_O_22_P = 1026.20 found [M − H]^−^ = 1025.28; [MNa − H]^−^ = 1047.09	13.4	0.55 ± 0.05	85.6
**8**	Calcd. for C_50_H_43_O_22_P = 1026.20 found [M − H]^−^ = 1025.11; [MNa − H]^−^ = 1048.99	14.7	0.34 ± 0.07	81.2
Silibinin (**1**)	–	–	1.40 ± 0.06	–
Quercetin	–	–	0.18 ± 0.01	–

^a^ For analysis conditions see Section 3.8.

**Table 2:** Spectroscopic characteristics and solubility of the silibinin (**1**) and the dimers (**6**–**8**) at pH = 6.5.

Compound	$\varepsilon_{288nm}$ (M^−1^∙cm^−1^)	Suspended in 10 mL	Theoretical Concentration	Abs_288 nm_ after Filtration	Solubility in H_2_O
**1**	22,158 (CH_3_CN) 19,469 (H_2_O/CH_3_CN, 98:2)	0.44 mg	91.2 µM *	0.018	≤0.9 µM (0.4 mg·L^−1^)
**6**	9100 (H_2_O)	0.10 mg	19.5 µM	0.18	≥19.5 µM (20.3 mg·L^−1^)
**7**	14,435 (H_2_O)	0.10 mg	19.5 µM	0.30	≥19.5 µM (20.3 mg·L^−1^)
**8**	18,536 (H_2_O)	0.10 mg	19.5 µM	0.35	≥19.5 µM (20.3 mg·L^−1^)

* The solution was saturated and the suspension was filtered before of the analysis.

**Table 3:** Singlet oxygen steady state concentrations [${}^{1}O_{2}$] and second order rate constant ($k_{{}^{1}O_{2},X}^{II}$ ) between singlet oxygen and silibinin (**1**) or dimers (**6**–**8**) in THF and H_2_O solutions.

Compound	THF		H_2_O	
	[${}^{1}O_{2}$]_SS_ (M)	$k_{{}^{1}O_{2},X}^{II}$ (M^−1^s^−1^)	[${}^{1}O_{2}$]_SS_ (M)	$k_{{}^{1}O_{2},X}^{II}$ (M^−1^·s^−1^)
**1**	3.79 × 10^−13^	6.1 ± 0.4 × 10^7^	-	-
**6**	-	-	4.07 × 10^−13^	6.8 ± 1.2 × 10^7^
**7**	-	-	4.07 × 10^−13^	5.4 ± 0.3 × 10^7^
**8**	-	-	4.07 × 10^−13^	4.0 ± 0.2 × 10^7^

**Table 4:** Second order rate constant ($k_{{\mathrm{HO}}^{•},X}^{II}$) between hydroxyl radical and **1** or dimers **6**–**8** in H_2_O/CH_3_CN (9:1) solutions.

Compound	$k_{{\mathrm{HO}}^{•},X}^{II}$ (**M^−1^·s^−1^**)
**1**	8.26 ± 1.64 × 10^9^
**6**	6.73 ± 0.32 × 10^9^
**7**	1.52 ± 0.08 × 10^10^
**8**	≥2.0 × 10^10^
